# Silent versus Reading Out Loud modes: An eye-tracking study

**DOI:** 10.16910/jemr.14.2.1

**Published:** 2021-10-21

**Authors:** Ioannis Smyrnakis, Vassilios Andreadakis, Andriani Rina, Nadia Bοufachrentin, Ioannis M. Aslanides

**Affiliations:** Hellenic Mediterranean University, Greece; Optotech Ltd., Greece; Harvard Medical School, USA; Brigham and Women’s Hospital, USA; Jamaica Plain VA Hospital, USA; University of Tübingen, Germany; MGH Inst. of Health Professions, USA; Hellenic Mediterranean Univ., Greece; Special Educator, Greece; Emmetropia Eye Institute, Greece; Wenzhou Medical Univ., China

**Keywords:** Eye movement, eye-tracking, dyslexia, reading problems, silent reading, loud reading

## Abstract

The main purpose of this study is to compare the silent and loud reading ability of typical and dyslexic readers, using eye-tracking technology to monitor the reading process. The participants (156 students of normal intelligence) were first divided into three groups based on their school grade, and each subgroup was then further separated into typical readers and students diagnosed with dyslexia. The students read the same text twice, one time silently and one time out loud. Various eye-tracking parameters were calculated for both types of reading. In general, the performance of the typical students was better for both modes of reading - regardless of age. In the older age groups, typical readers performed better at silent reading. The dyslexic readers in all age groups performed better at reading out loud. However, this was less prominent in secondary and upper secondary dyslexics, reflecting a slow shift towards silent reading mode as they age. Our results confirm that the eye-tracking parameters of dyslexics improve with age in both silent and loud reading, and their reading preference shifts slowly towards silent reading. Typical readers, before 4th grade do not show a clear reading mode preference, however, after that age they develop a clear preference for silent reading.

## Introduction

A useful ability that a person develops from an early age, is reading, which is a result of several cognitive skills working together in a coordinated, well-integrated fashion. When we talk about cognitive skills, we are referring to the mental processes that our brain undergoes, in order to be able to comprehend, organize and store information, which at some point we will retrieve and use; these cognitive skills are essential for reading and can have a direct impact on the individual’s life (i.e. academic performance, career and more). Examples of those skills can be the visual scanning, selective focusing, retrieving information from lexical storage and short-term memory (14). People may read silently or aloud. During silent reading, the person reads without any vocalization, whilst during loud reading is required the pronunciation of phonemes to be synchronized with the continuous visual scanning of the text. In both, the reader needs to complete three language processing levels; phonological decoding, morphological decoding and semantic decoding (giving meaning to the decoded representation), plus the vocalization for the loud reading.

Growing up, typical readers will first develop decoding ability in the recognition of well-known morphemes, and then develop word expectation ability (12). In order to do the decoding, our brain uses paths that are involved in spoken language, such as the Broca’s area and the left Brodmann’s area (20).

Auditory and visual discrimination, along with the ability to move smoothly from words to paragraphs or lines, the processing speed and the ability to move from the reading word-by-word stage to make groups out of them and sentences at the end, are important elements for comprehension (38). Comprehension is the main reason why we learn to read but often it’s not necessarily achieved in all occasions, not even from fluent readers (33).

After the student has learned the spelling rules and is familiar with the lexical complexity, he/she can read the word representation stored in the memory (6). These complex processes usually become automatized over time (6). However, younger or less skilled students, regardless of age, often put most of the decoding (25). In most cases, as students grow older, they adopt lexical or sub-lexical strategies (28). When using a lexical strategy, the reader recognizes the word as a unit related to a meaning. Hence, the student bases his/her reading fluency in word familiarity; the more familiar he/she is with a variety of words, the more fluent. On the other hand, when using a sub-lexical strategy, the person perceives the word partially. Consequently, the longer the word is, the more difficult it becomes for students.

Language orthography differences are key in investigating difficulties in reading (18; 21; 35). Spelling is divided into two categories, with transparency being an important determinant criterion. In transparent-shallow languages, also called shallow orthographies, such as Spanish, Italian, Finish and Greek, spelling reflects the phonology. So, letters have the same pronunciation, regardless of the word they appear in. In non-transparent languages, also named deep orthographies, such as English, French, Thai and Hungarian, symbols-graphemes might map to different sounds in the various letter combinations-words.

According to the DSM-5 (3) criteria, specific learning disorder is a neurodevelopmental disorder with a biological origin that is the basis for abnormalities at a cognitive level that are associated with the behavioral signs of the disorder. The biological origin includes an interaction of genetic, epigenetic, and environmental factors, which affect the brain's ability to perceive or process verbal or nonverbal information efficiently and accurately. Brain differences impact the rate of processing visual information per second (18). Phonological decoding, spelling, accuracy and fluency of word recognition are influenced by neurodevelopmental differences (17; 30; 43) affecting the ability to read, write and comprehend. What is more, phonological and visual attention deficits are related to impairments in short-term memory (44) that may also affect reading. Typical students, while reading, use their memory to give meaning to what they decode and to comprehend sequenced phrases, sentences and the whole text. Over time, these processes are automatized and reading becomes more fluent. Contrary to typical readers, dyslexics face difficulty in automatizing reading and developing a reading strategy. This may, in part, be due to their struggle in processing information stored in their short-term memory due to the difficulty they face in the phonological decoding phase of reading. The term “dyslexia”, which is the most common specific learning disorder, has been controversial and an issue for debate among educators, cognitive scientists and neurologists for years. Some, do not even believe dyslexia exists (26), for others it is a specific neurological disorder, while others refer to dyslexia as an “umbrella-term”, which includes all the reading disabilities (41). Specifically, due to the use of dyslexia as an “umbrella-term”, the psychologists were trying to find ways to diagnose dyslexia by showing possible differences between what they expected from the pupils to read and what was the reality of this; the use of IQ tests (a combination of Verbal, Performance and Full-scale IQ tests) were used as assessment tools for children (including preschoolers) and adults. This effort though failed to identify qualitative differences between the reading in children with general learning difficulties and those with dyslexia, and that led to a move away from an ‘IQ-discrepancy’ definition (41). In this study, by using the term dyslexia, we refer to developmental dyslexia. Previous studies have argued for the differentiation between developmental dyslexia and as Snowling et al. call, “acquired” dyslexia (40) or (according to others) alexia (1). Alexic patients were previously literate but due to an acquired event (usually a stroke or other brain injury), are unable to read (1) and/or comprehend letters, words and/or sentences. When individuals show these characteristics from an early age, their dyslexia is called “developmental” (43; 44).

The diagnosis of dyslexia requires persistent symptoms to be present for more than 6 months. If so, the symptoms usually persist during adulthood (3). Symptoms should be present in more than one environment (home, school, etc.) and affect the individual’s performance in these surroundings (3). Although there is no correlation between dyslexia and lower than normal intelligence (3), in school, dyslexic students struggle to keep up with their classmates when written tasks are involved, and this often does not reflect their true potential. Hence, their overall academic performance may be lower than what it could potentially be. In Greece, the diagnosis of dyslexia requires a discrepancy between the IQ score and level of academic performance; that is that the person’s IQ score should be average or higher than their academic performance indicators (34).

There are multiple studies showing that dyslexia does not occur due to oculomotor impairments (27; 37). Nevertheless, tracking the eyes during reading yields plenty of information for classifying each individual’s reading profile. Using the eye-tracking technology can help us detect and record fixations and saccadic movements. Fixations are intervening times during which the eye stays on target, while not moving or blinking and are of average duration of ~200ms (36; 37). Saccades refer to the rapid eye movements that occur between sequential fixations (5; 27; 36). Parameters extracted during eye-tracking can be separated into two groups, “non word-based” and “word-based”. There is a lot of prior research that focus on “non word-based” parameters like fixation number, saccade length etc. The “word-based” parameters, described in detail in a later section, are parameters that generate information about how the individual interacts with a specific written text while reading, and decodes a specific word or a group of words.

Dyslexic readers and typical readers exhibit significant differences in eye-tracking parameters during reading (29; 45; 48), accounting for how eye movement parameters might be affected by the child’s development as well as the child’s will and effort to cooperate (36). Variations have also been observed among dyslexics and typical students, in loud versus silent reading. In general, loud reading is a more complicated process because it allocates more cognitive resources for pronunciation, intonation and word stress (19). However, some studies claimed that loud reading favors comprehension more than reading silently (8; 19).

De Luca et al. study found that both typical readers and readers with dyslexia spend more time reading out loud than silently (14). Nevertheless, particularly for the dyslexics, who were in general slower than the typical readers in both modes (14), reading was effortful and time consuming (18), as they struggle with the automaticity of reading and the reduced fluency and they display a deficit in reading speed and accuracy depending on word length (6; 42).

Buswell (9) highlights that the time gap between the eye movement and the voice during loud reading is larger in dyslexic students. De Luca et al. (14) suggest this is due to dyslexic individuals having more frequent long pauses and regressions than controls. Fairbanks (16) points out that the hesitation caused by insufficient decoding and intelligibility in the process of loud reading can cause an eye-speech gap.

The purpose of this study is to compare the silent versus the loud reading ability of typical students and students with dyslexia in elementary, secondary and upper secondary school grades. The comparison is carried out using the eye-tracking technology. The eye movement parameters extracted from silent and loud reading, namely reading speed, fixations number, mean fixations duration, mean saccade length, 25, 50 and 75 percentiles of saccade length distribution, not-fixated words, multiple fixated words, gaze duration on group of words and number of backward refixations, were used to evaluate and point out the reading mode in which each student performed better. The results of the comparison of silent and loud reading could contribute to the improvement of reading assessment, and hence to the planning of individualized, more efficient, intervention.

## Materials and Methods

### Participants

One hundred fifty-six (156) students (ages: 8-17.3, 74 girls / 82 boys, from 3^rd^ grade of the primary school to 11^th^ grade of the upper secondary school) were recruited and they were all native Greek speaking students in Greece. Twenty-six (26) of them were rejected due to unreliable eye movement recording or lack of cooperation with the researcher (the research team was blind to the diagnosis until rejection).

The remaining 130 students were divided in two populations – (a) the Dyslexic population: 61 participants (20 girls, 41 boys) officially diagnosed with dyslexia by the Greek governmental agency and (b) the Typical readers: 69 participants (40 girls, 29 boys) randomly recruited among students, who were further assessed by a special educator to be cleared of any reading or learning difficulty. The dyslexic population was diagnosed by the Greek Centers of Educational and Counselling Support (in Greek K.E.S.Y.). This diagnosis involves assessments in word decoding, fluency, syntax, grammar and comprehension. Furthermore, participants were examined psychiatrically and screened for more serious disorders (lower than normal intelligence etc.). In Greece, there is no universally accepted standardized test that is used for dyslexia diagnosis. No criteria based on the severity or the precise form of the disorder were applied. The typical readers were recruited among school students that did not have any reported reading difficulties as judged by their school teacher. All these students were further screened by a special education teacher for obvious reading difficulties. No student was disqualified by this screening. All participants had normal or corrected-to-normal vision and normal hearing levels. Participants were separated into three groups according to their school grade. Group A included students of the 3^rd^ and the 4^th^ grade of the primary school, Group B included the 5^th^ and the 6^th^ grade students of the primary school and Group C included students from the 7th to the 11th grade of the secondary and upper secondary school (Table 1). We chose these specific age groups in order to be able to assess how students improve in reading over the years.

**Table 1:** Participants’ numbers in each school grade and each group created for analysis purposes.

School Grades	Typical readers	Dyslexic readers
Grade 3	20	10
Grade 4	16	15
Grade 5	12	11
Grade 6	9	15
Grade 7	2	7
Grade 8	5	2
Grade 9	0	1
Grade 10	4	0
Grade 11	1	0
		
Groups created for analysis		
Group A (3^rd^ & 4^th^ grade)	36	25
Group B (5^th^ & 6^th^ grade)	21	26
Group C (7^th^ - 11^th^ grade)	12	10

Written informed consent was obtained from the parent or legal guardian, and the child’s consent was obtained before the test. The study conformed to the tenets of the Declaration of Helsinki and ethical approval was provided by the review board of Optotech Eye Tracking Ltd.

### Stimuli

#### Text stimuli

Participants were asked to read silently and out loud a text in the Greek language, while their eye movements were recorded. The text was written by a special education teacher in order to be appropriate for all age groups and was the same for all participants. It had 181 words, most of which are multi-syllable, it included high and low frequency words chosen from the teacher’s books of elementary school grades (all schools are using the same books across Greece), and its content was of middle primary school difficulty. The statistics of the text are shown in Table 2. The text was written in black Courier New font presented on a grey background. The font size was 30pt, mono-spaced with in line space 2.3 lines. The text had 28 lines, divided in five screens (6 lines were presented on each of the first four and 4 in the last one). For both reading tasks, in order to move to the next page, the children had to press the ‘Space Bar’ key on the keyboard.

**Table 2:** Detailed statistics for the text. The text was written by a speech pathologist.

Total word count:	181
Unique words:	114
Total number of characters:	1168
Number of characters without spaces:	986
Average characters per word:	5.44
Average syllables per word:	2.37
Sentence count:	13
Max sentence length (words):	8
Min sentence length (words):	2

Five comprehension questions were asked after the full reading of the text and were answered orally with a “YES” or “NO” and they were automatically stored in the database. For missing or incorrect answers, the score was 0 points, and for correct answers, the score was 1 point. Therefore, the highest composite score was 5 points. The purpose of these questions was not to strengthen understanding, but to increase the probability for participants to read the whole text. The number of correct answers is not used to reject participants and is not a part of the evaluation. The participants were aware of the 5 comprehension questions from the start of the assessment.

### Apparatus

Eye movements were recorded using a Tobii 4C eye-tracker (47). This eye-tracker has 90Hz sampling rate, 50cm-90cm eye capturing distance and is easily attachable/detachable on the laptop’s screen. To run the experiments, a Dell laptop was used with an Intel i7 processor and a 15.6’’ screen size. The display resolution of the monitor was set to 1366 × 768 pixels with a refresh rate of 60 Hz. No head-rest was used. This eye-tracker model supplements eye tracking with head tracking, hence it is possible to project the visual axis to the computer screen without the use of a headrest.

The fixations identification was performed through clustering. When the eyes stayed within a radius of 90 pixels from the centroid of gazes cloud (about 4 characters in this setup), for a time greater than 90ms, then that reflects to a fixation with a duration equal to the time of gazes within this radius. The values of the radius and the time threshold were chosen so that the overall number of fixations was stable under small perturbations of both the radius and the time threshold. Regarding saccades, in order to ensure that the movement between two consecutive fixations was a true saccade, a speed limit of 60 deg/sec was applied.

### Procedure

**Figure 1:**
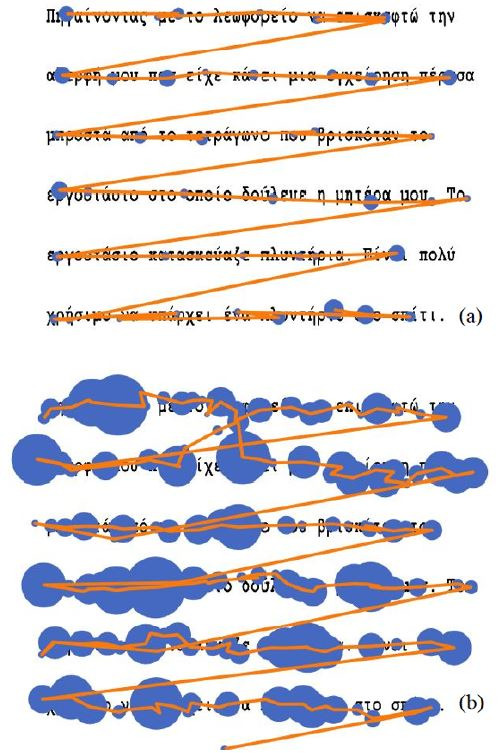
Reading paths from a typical reader (a) and a dyslexic reader (b). The blue circles are the fixations and the orange lines are the saccadic movements. The bigger the circle, the longer the fixation. The reader with dyslexia exhibits longer fixations, shorter saccades and more regressions (back and forth movements).

Before performing the main task, a basic vision screening test was performed by an ophthalmologist, which was considered a prerequisite for inclusion in the study. The students were sitting in a quiet room for testing. In front of them, there was a laptop with a viewing distance of about 50cm-60cm. Instructions were given to them by a researcher, who was present for the duration of the session. Testing was lasting approx. 15 mins. Figure 1 a &b exhibits the reading paths of a typical and a dyslexic student as it was captured real-time by the eye-tracker.

The main task consists of three parts: calibration and validation, silent reading and loud reading part. For all parts, the students did not have to talk to the researchers to eliminate their movements while recording their eye movements.

### Calibration and validation stimuli

A series of seven, different, blue spinning dots were displayed in succession, in known coordinates symmetrically positioned on a matrix grid. After the calibration was complete, a validation process followed. Calibration was typically performed once at the beginning of the session. Though, to maintain and increase the data accuracy, another safety valve was introduced before and after each text reading. This time, a series of five small red target-shaped dots were displayed consecutively. A smaller red dot at the center of the target served as a fixation point. The participants were instructed to follow the stimulus, only with their eyes, while trying to fixate at the center of the target. A validation point was considered successful if 90% of the gazes were located inside a circle that had as a center the red dot and radius equal to 90 pixels. Then, the participants were proceeding to the reading part. In case the validation was unsuccessful, another calibration process was performed. The last validation screen was for offset calculation purposes (if needed), in case the participant moved from his/her original position.

### Silent reading and reading aloud

After a successful calibration and validation, the reading protocol was applied as described below:

*Silent reading:* Participants were required to read a Greek text silently at their own pace, because the purpose of the task was not to read quickly, but to read accurately. At the end of reading, they had to answer five comprehension questions.

*Loud reading:* The last thing the participants had to do was to read the same text as before, but this time out loud. Contrary to the silent reading, there were no comprehension questions.

## Results

The students’ eye movements were analyzed while they were reading silently and loud. For each eye-tracking parameter analyzed, two values were extracted: one for silent and one for loud reading. Our analysis is divided into three parts: In the first part the improvement of reading speed is examined as a function of school grade, for grades 3 to 8. Population sizes did not permit us to reliably extend this to grades 9 through 11. In the second part, the analysis is carried out in three groups (Group A, 3^rd^-4^th^ grade, Group B, 5^th^-6^th^ grade, and Group C, 7^th^-11^th^ grade). This grouping was performed in order to have relatively homogeneous groups comprised of the biggest possible population. Groups A and B are comprised of two grades each to maintain a uniformity in reading skills, and Group C involves five grades, since by that level onwards, students are expected to have mastered reading reasonably well and to have similar reading skills. The purpose of this part of the analysis is to examine the difference in parameter values between silent and loud reading. To assess the significance of this difference, it is normalized by the standard deviation. This is called “Asymmetry” and is explained in detail later. In the third part, the analysis is also performed in the Groups A, B and C, but this time the average values of the parameters are evaluated and their variation in correlation to the groups created is studied.

### Part 1: Reading speed in relation to age

**Figure 2:**
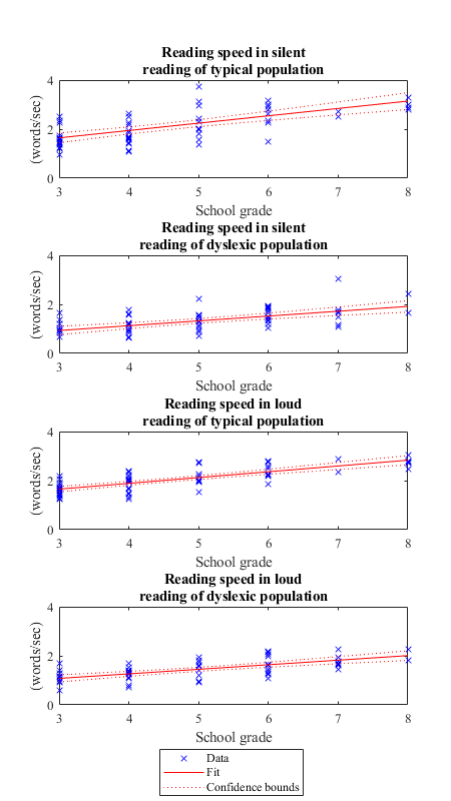
Reading speed vs. school grade for silent and loud reading for both populations. Red lines represent linear fits. Dotted red lines represent the 95% confidence intervals of the fitting parameters. The slope of the linear fit of the reading speed vs class number for typical population is higher than the corresponding slope for the dyslexic population in silent reading (p 0.03) and loud reading (p 0.1), meaning that dyslexic population exhibits slower progress on average than the control population in the age group from 3^rd^ to 8^th^ grade.

Reading speed is one of the eye-tracking parameters that we evaluate in the present study. This analysis is performed separately for each school grade from 3^rd^ to 8^th^ grade. On average, the dyslexic population exhibits slower progress than the control population as students grow older. The slope of the linear fit for the control population is higher than the corresponding slope for the dyslexic population in both silent (p=0.03) and loud (p=0.1) reading (Fig. 2). In Figure 2, it is shown that the average reading speed between the two groups differs from the very beginning, i.e. from Grade 3 of our analysis. Even though the reading speed improves in both populations, there is still a steady difference between controls and dyslexics.

### Part 2: Asymmetries of the parameters

Asymmetry between silent and loud reading parameters is the difference between the parameter values normalized by the standard deviation of the difference. The asymmetries were evaluated separately for the control group and the dyslexic group. The asymmetries are calculated using the following formula: $A=\pm \frac{x1-x2}{\sqrt{\sigma_{1}^{2}+\sigma_{2}^{2}}}$, where A is the asymmetry of the parameter, x_1_ is the value of the parameter in silent reading, x_2_ is the value of the parameter in loud reading and σ_1_ and σ_2_ are the standard deviations of x_1_ and x_2_ respectively, as computed from the relevant population. The value of A was calculated for each participant. The mean value of A (i.e.‾A) was calculated separately for the typical readers and separately for the dyslexic readers, and the plus-minus sign symbol was chosen so that a positive difference shows better parameter value in loud reading and a negative difference shows better parameter value in the silent reading (for the population). The error bars were calculated by the following formula: $\pm e=\pm \frac{\sigma_{Α}}{\sqrt{Ν}}$ , where e is the error, σ_Α_ is the standard deviation of the asymmetries on each population and N is the number of participants for each population.

### Eye-tracking parameters examined

The parameters for the asymmetry analysis were selected due to their discriminative power among typical and dyslexic participants. We divided them in two categories: “non word-based” and “word-based”. The “non word-based” parameters were named as such because they are not related to specific words in a text. These parameters can be measured with the use of eye-tracking technology whatever the stimulus is (e.g. text, picture etc.). The other set of parameters, the “word-based” ones, were named as such because they are related to the words of the text and are affected by characteristics like the length or the familiarity of words.

Statistical analysis was performed using t-test when appropriate, because of the small group populations involved. P-value less than 0.1 was considered as statistically significant.

The “non word-based” parameters analyzed were: reading speed (R.S.), fixations number (F.no), mean fixation duration (M.F.D.), mean saccade length (M.S.L.) and 25%, 50% and 75% percentiles of saccade length. Reading speed is calculated by the number of words read per second. Fixations number counts the total number of fixations across the text, i.e. how many times the readers stops so that brain can assimilate the information. Mean fixation duration is the average duration of fixations in total across the whole text. A high mean fixation duration indicates difficulty in decoding. Saccade length measures the average distance between two consecutive fixations, in pixels (in our apparatus, 23 pixels equal to approximately 1 letter). Long saccade length indicates great reading fluency and confidence, while short saccade length indicates syllabic reading and difficulty. To monitor the whole distribution of saccade lengths, apart from the mean saccade length the 25%, 50% and 75% percentiles were derived.

The “word-based” parameters analyzed were: words with no fixations on them (N.F.W.), words that were multiple fixated (M.F.W.), mean gaze duration (first visit time) on words with 6-7 characters (6-7 char), mean gaze duration on words with 8+ characters (8+ char), and total number of backward within word refixations (B.RF.). Not-fixated words are the total number of words that do not have any fixations. A large number of words without a fixation during reading indicate word predictions (guessing ability based on context), or use of peripheral vision. Both of these skills are developed in fluent readers. Multiple fixated words are the words that have more than a single fixation, either during the first visit of the word or after moving to another word and return. Multiple word fixations show either difficulty in decoding (if in first visit) or difficulty in comprehension (if they result from moving forward and backward). Mean gaze duration of words with specific number of characters (6-7 characters and 8+ character words) is calculated as the mean time of first visit on each word of that group. A low mean gaze duration is related to higher decoding ability. Groups of words with less than 6 characters were discarded from this analysis as they provided no supplementary information. The last parameter analyzed was Backward Refixations Number which is the total number of backward movements in a word. Many backward refixations while reading is a common characteristic of dyslexic readers.

### Asymmetry directionality

The asymmetries of the parameters Reading Speed, Mean Saccade Length, 25, 50 and 75% percentiles, and Not-Fixated Words are calculated by the value of each parameter during loud reading minus the value of each parameter during silent reading, normalized by the standard deviation of the difference. High values of these parameters, based on our analysis, indicate fluency in reading.

The asymmetries of the parameters Fixations Number, Mean Fixations Duration, Multiple Fixated Words, gaze duration of 6-7 char. words, gaze duration of 8+ char. words and Backward Refixations are calculated by the value of each parameter during silent reading minus the value of each parameter during loud reading, normalized by the standard deviation of the difference. Low values of these parameters, based on our analysis, indicate fluency in reading.

The difference in asymmetries calculations comes from the fact that we decided positive asymmetry to indicate “preference” in loud reading and negative asymmetry to indicate “preference” in silent reading. By using the word “preference”, we mean that between the two reading modes, the “preferred” mode is the mode were the participants had reduced number of fixations, smaller saccadic movements, larger number of not-fixated words, smaller number of multiple fixated words, lower gaze duration on words groups with words with 6+ characters, reduced number of backward refixations and were reading faster in comparison with the other mode.

**Figure 3:**
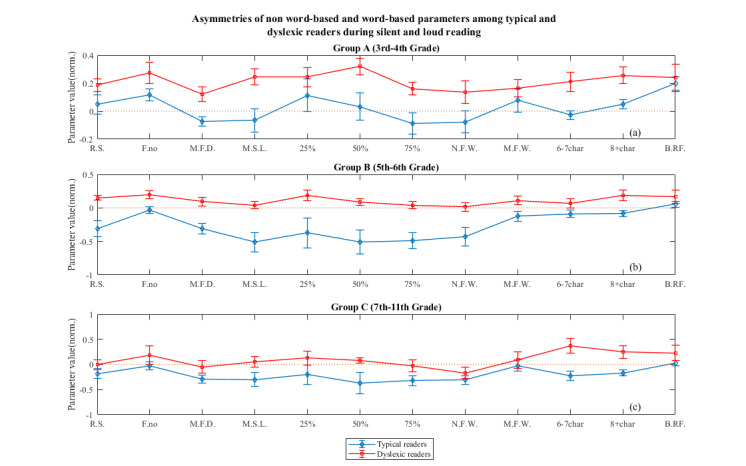
Asymmetries for all parameters, separately for each group during reading. The blue line depicts the asymmetries for the typical population and the red line depicts the asymmetries for dyslexic population. A positive asymmetry value in a parameter is translated in better reading performance in loud reading in comparison to silent, while a negative asymmetry value is translated in better reading performance in the silent reading in comparison to loud. An asymmetry close to 0 (dotted line) is translated in equal reading performance in both reading modes. The error is calculated as the standard deviation of the asymmetries on each population divided by the square root of the number of participants on each population (typical and dyslexic readers). R.S.=reading speed, F.no=fixations number, M.F.D.=mean fixation duration, M.S.L.=mean saccade length, 25%, 50%,75%=25,50,75 percentiles, N.F.W.=words with no fixations, M.F.W.=words that are multiple fixated, 6- 7char=gaze duration on 6-7char. words, 8+char=gaze duration on 8+ char. words and B.RF.=backward refixations.

*Typical Group A:* As we can see on Figure 3, the asymmetries for this group are positive at significance level 0.1 (supplementary file, Table 1 and Table 2) for parameters Fixations Number, 8+ char (gaze duration) and Backward Refixations. They are negative at significance level 0.1 (supplementary file, Table 1 & Table 2) for the parameter Mean Fixations Duration. The rest of the parameters have asymmetry close to 0. Thus, in this group, typical readers exhibit approximately the same reading skill in both reading modes, showing no preference in either of them.

*Dyslexic Group A:* Contrary to the typical readers of Group A, the asymmetries of dyslexics in Group A are significantly positive (supplementary file, Table 1 & 2), showing better reading skill in loud reading in all the parameters. Hence, it seems that dyslexics of this age read better if they can hear themselves read instead of reading to themselves. What is furthermore important is that the asymmetry of dyslexics is higher than the asymmetry of typical in almost all parameters, except marginally 25% percentile, Multiple Fixated Words and Backward Refixations, where the asymmetry of dyslexics is still higher, but not significantly.

*Typical Group Β:* The asymmetries for the typical readers of this group are significantly negative (sup.file, Table 1 & Table 2) for all parameters except Fixations Number and Backward Refixations where it is almost zero. This result indicates an overall reading preference for silent reading. The parameters Fixations Number and Backward Refixations that had positive asymmetry in Typical Group A, have now asymmetry close to zero which is translated to no preference to either reading mode.

*Dyslexic Group Β:* In dyslexic readers of this Group, the asymmetry in most parameters is significantly positive (sup.file, Table 1 & Table 2), which still shows a preference toward loud reading, although the trend is less prominent than for the dyslexic group of lower age (Group A). The asymmetries are significantly positive for Reading Speed, Fixation Number, Mean Fixation Duration, 25% Percentile, 50% Percentile, Multiply Fixated Words, 8+ Words.

*Typical Group C:* The asymmetries for the readers of this group are significantly negative (sup.file, Table 1 & Table 2) for all parameters, meaning that they read better when they read silently, except Fixations Number, 25% Percentile, Multiple Fixated Words and Backward Refixations where it is almost zero. This seems to confirm the trend that appeared in Typical Group B which is that typical readers seem to prefer to read silently as they grow older.

*Dyslexic Group C:* For dyslexic readers of Group C, Not-Fixated Words is significantly negative (sup.file, Table 1 & Table 2), Gaze dur. 6-7 char, 8+ char. and Backward Refixations are significantly positive (sup.file, Table 1& 2), while the rest asymmetries are close to zero or negative. That means that there is no preference for either of the reading modes. But, taking into account the clear preference in loud reading showed in younger ages (Group A), it seems they tend to slowly adapt to the silent reading mode as they grow older. Note that in all groups, asymmetries of dyslexic population on all parameters have higher average values than typical population, suggesting that dyslexic readers adapt slower to the silent mode of reading than typical readers.

### Part 3: Analysis on parameters’ values

In the third part, the analysis is performed again in Groups A, B and C, but this time the average values of the parameters are evaluated. Their differentiation in relation to the age groups is studied. Furthermore, the difference of silent and loud reading parameters is compared with the difference of typical and dyslexic reading parameters. This comparison is performed by computing the ratios $d_{T}=\frac{\left|\bar{S_{T}}-\bar{L_{T}}\right|}{D}$ and $d_{D}=\frac{\left|\bar{S_{D}}-\bar{L_{D}}\right|}{D}$, where d_T_ and d_D_ are the ratios of the typical and dyslexic population respectively, S_T_ and S_D_ are the mean parameter values of silent reading of typical and dyslexic readers respectively, L_T_ and L_D_ are the mean parameter values of loud reading of typical and dyslexics readers respectively and finally D is calculated by $D=|\frac{\bar{S_{T}}+\bar{L_{T}}}{2}-\frac{\bar{S_{D}}+\bar{L_{D}}}{2}|$ which is the difference between typical and dyslexic parameter values.

Reading Speed: the parameter’s values increase with age for both typical and dyslexic readers, in both reading modes. For all age groups, the ratios d_T_ and d_D_ between silent and loud reading values within typical population and within dyslexic population is much smaller than the average difference between control and dyslexic population ($d_{T}\;\text{ and }\;d_{D}\ll 1$, supplementary file, Table 3). Another point worth mentioning is that moving from Group A to Group C, i.e. as students grow older, the difference D in average reading speed between control and dyslexic readers increases for the age groups considered (supplementary file, Table 4).

**Figure 4:**
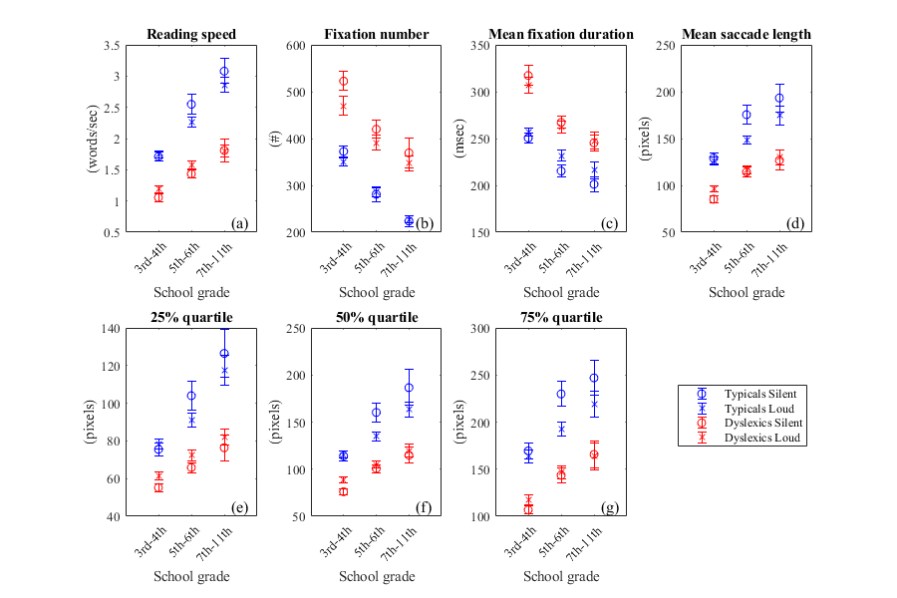
Values of “non word-based” parameters of typical and dyslexic population, during silent and loud reading. Each population was divided in three groups: Group A: 3rd and 4th grade, primary school. Group B: 5th and 6th grade, primary school. Group C: 7th to 11th grade, secondary & upper secondary school. The error bar is the standard error of the mean. The parameters were: reading speed(a), the total number of fixations(b), the average duration of fixations(c), the average length of saccades(d), and the 25%(e),50%(f) and 75%(g) percentiles of saccades. The parameters were: reading speed(a), the total number of fixations(b), the average duration of fixations(c), the average length of saccades(d), and the 25%(e),50%(f) and 75%(g) percentiles of saccade length.

Fixation number: High values of the parameter translate to difficulty in reading and it is clear that the dyslexics have higher values than controls. (Fig. 4b). The difference of silent and loud reading values of the parameter within each population is smaller than the average difference between the two populations ($d_{T}\;\text{ and}{\;\text{ d}}_{D}\ll 1,\;$supplementary file, Table 3). In addition, there is a rapid drop in the fixation number with age for both control and dyslexics from Group A to Group B, although it slows down from Group B to Group C. The difference D between the two populations is kept almost the same (supplementary file, Table 4).

Mean fixation duration: Regarding the values of the parameter, control population in all groups consistently has lower parameter values (shorter) than dyslexics which suggests that control participants decode faster than dyslexics (Fig. 4c). Again, $d_{T}\;\text{ and}{\;\text{ d}}_{D}\ll 1$, suggesting that the difference of silent and loud reading values of the parameter within populations is much smaller than the average difference between the two populations (sup.file, Table 3). The difference D of average values of silent and loud readings between the two populations is bigger in Group A, and as students grow older, this difference is reduced (sup.file Table 4). Typical readers reach the value of approx. 200-220ms much faster than dyslexics. This is close to the mean fixation duration during silent reading as reported in (37).

Mean saccade length and percentiles: Regarding the parameter values, low values indicate lack of reading fluency. The mean saccade length of typical readers is clearly smaller than of dyslexics. Here, $d_{T}\;\text{ and }\;d_{D}\ll 1\;$for typical Group A and dyslexic Group B&C, however $d_{T}\;\text{ and }\;d_{D}\;$are of the order 0.2-0.6 for typical Group B&C and dyslexic Group A. (sup.file, Table 3). In addition, it is worth noting that the difference between typical and dyslexic readers is higher in silent reading than in loud reading (Fig. 4 d to g).

**Figure 5:**
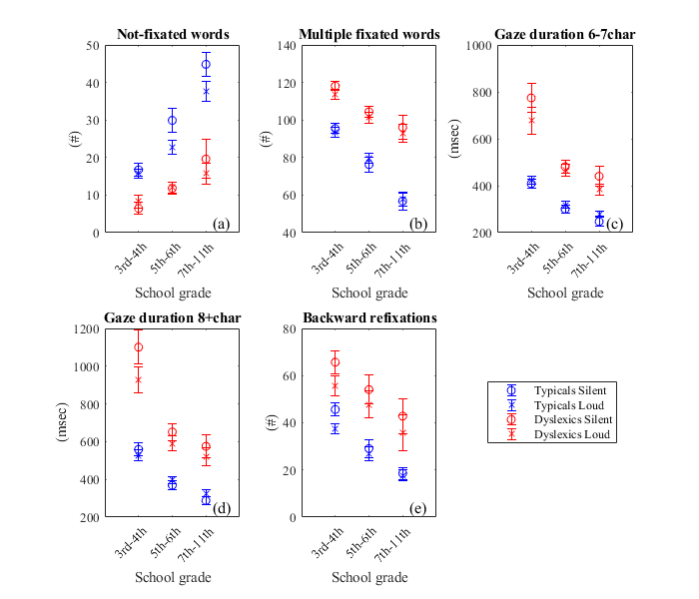
Values of “word-based” parameters of typical and dyslexic population, during silent and loud reading. Each population was divided in three groups: Group A: 3rd and 4th grade, primary school. Group B: 5th and 6th grade, primary school. Group C: 7th to 11th grade, secondary & upper secondary school. The error bar is the standard error of the mean. The parameters were: number of words with no fixation on them(a), words with multiple fixations on them(b), gaze duration of words with 6 and 7 characters(c), gaze duration on words with 8+ characters(d), and the total number of backward refixations(e).

Not-fixated words: Regarding the values of the parameter, control population in all groups consistently has significantly higher parameter values than the dyslexic population (Fig. 5a). In addition, $d_{\text{T }\;}\;\text{ and }\;d_{D}\ll 1$ for all age groups, except typical Group B which is of value 0.5 (sup.file, Table 3). As students grow older, the number of not-fixated words increases (expressing improvement) for both control and dyslexics. However, this rise is slower in dyslexics, hence the difference D between control and dyslexics becomes wider (sup. file, Table 4).

Multiple fixated words: High values of the parameter translate to difficulty in reading and clearly dyslexics have higher parameter values than typical readers. The ratios $d_{T}\;\text{ and }\;d_{D}\ll 1$ (sup. file, Table 3) in all groups. Moreover, the number of multiple fixated words decreases with age, however this drop is more rapid for typical students of Group C (Fig. 5b). The difference D between the two populations is getting bigger as students grow older (sup. file, Table 4).

Mean gaze duration of words with specific number of characters (6-7 characters and 8+ character words): In all groups, $d_{T}\;\text{ and }\;d_{D}\ll 1\;$(sup.file, Table 3). For both parameters and for both silent and loud reading, the difference D between typical and dyslexic populations is more prominent for Group A students (sup. file, Table 4). This difference is reduced rapidly with age (sup. file, Table 4), which means that these parameters have high discriminative value in younger ages but lower discriminative value as students grow older (Groups B&C, Fig. 5 c & d).

Backward refixation number: In both control and dyslexic students, the number of backward refixations decreases with age. This parameter value is related to difficulty in decoding segments of a word (graphemes). The ratios $d_{T}\;\text{ and }\;d_{D}$ are below 0.1 typical Group B&C, and between 0.3 to 0.5 for the other groups (sup. file, Table 3). In addition, the difference D between the two populations is steady as it is almost the same in all age groups (sup. file, Table 4). One point worth mentioning is that in all age groups, during loud reading, the dyslexics have lower number of backward refixations, i.e. they don’t need to go back and forth as often as they do while reading silently (Fig. 5e).

## Discussion

There are multiple studies showing that dyslexia does not occur due to oculomotor impairments (27; 37) but also there are others which found a pattern of oculomotor anomalies in children with learning disabilities (including dyslexia) compared to typical readers (4; 7; 10, 11; 15; 39). Nevertheless, compared to typical readers, difficulty in decoding results in different eye-tracking reading paths for those with reading difficulties, such as dyslexia (29; 45; 48). These paths, might show that higher-level of attention is allocated on more basic oculomotor processes and probably this is what in turn leads to lower ability in understanding the words per se (22; 46).

In this study we focus on comparing silent and loud reading of typical and dyslexic students, as a function of age, showing which population prefers which mode of reading. We found that (a) typical students perform better in silent reading, especially in Groups B&C (5^th^ to 6^th^ and 7^th^ to 11^th^ grade), while dyslexics perform better in loud reading, especially for Groups A&B (3^rd^ to 4^th^ and 5^th^ to 6^th^ grade). The latter seems to be true for the English speaking population as well (29).

Typical readers’ preference may depend on the fact that, in loud reading, they need to complete several tasks at once, such us decoding, articulation and prosody (2; 45). Prosody includes features like pauses, rhythm, intonation, tone and quantity (vocal intensity and duration). For example, articulation and prosody seem to reduce the loud reading speed of typical students compared to their silent one. Nevertheless, it is surprising that the same steps of loud reading that make it difficult for control students to read aloud, seem to have the opposite effect on dyslexic students. This may be because articulation and prosody make loud reading a multi-sensory task, which enhances the ability of dyslexic individuals to correct themselves while reading and listening to themselves, which does not happen during the silent reading mode (2; 45).

Accurate eye-tracking is able to provide valuable data for any field of work in which visual stimuli are shown to students and they are required to evaluate them (27; 37). Having a number of eye-tracking parameters monitored, at all reading modes as a function of age (school grade) showed longer fixation-durations, increased number of fixations, shorter saccadic movements, lower reading speeds and a great number of multiple-fixation words for the dyslexic population; that revealed the struggle of this population when it comes to word-decoding and reading fluency. These findings are in alignment with prior studies (13; 21; 32). In addition, dyslexics put more effort to read, and have poor anticipation skills as evidenced by the high fixation number, the fewer not-fixated words and the higher number of backward refixations.

We also observed that typical readers tend to predict and avoid fixating on short words ₋ such as articles, conjunctions and words they are familiar with. The existence of a higher number of not-fixated words in typical population suggests that typical readers have a greater skill in predicting words during reading compared to dyslexic readers. This skill is based on the typical students’ previously established knowledge, which increases as students grow older, and to their ability to process fast the morphosyntactic features of the text and to comprehend what they read (23; 29; 31). Detecting words in the visual periphery may also play a role (24). Hence, as students grow older, the number of not-fixated words also rises. That is something that applies not only to typical readers, but also in dyslexic readers, although in dyslexics this rise is shorter. In typical Groups B and C, students have high word predictability (higher number of not-fixated words, Fig. 5a), thus they tend to read faster (high reading speed, Fig. 4a), as they do during silent reading in comparison to loud reading. This conclusion does not apply for the younger students of the typical population (Group A), and for young students with dyslexia (Group A&B), as they have approximately the same values in both reading modes. Nevertheless, as dyslexics students grow older (Group C) they also tend to avoid focusing on short words, just like the typical readers in Group B and C do.

A general conclusion of our study is that typical students prefer to read silently. This applies in younger ages, like the students in 3^rd^ to 4^th^ grade in our study, but not entirely. The reading preference of this group is less distinct. There are parameters like Fixations Number, 8+ char (gaze duration) and Backward Refixations which are statistically significant in favor of silent reading preference. But the rest of the parameters are either close to zero or positive, leading to no reading preference. This result can be attributed to the fact that at the beginning of the school, students learn to read orally rather than silently. Their first steps to silent reading, start on the 3^rd^ grade of primary school (in the Greek educational system). Hence, this unclear preference could be attributed to the fact that they are still learning to read silently.

As typical students grow older, they improve steadily and significantly their reading abilities in both loud and silent reading, mastering their abilities in both reading modes and showing their clear preference for silent reading at older ages. Although the eye-tracking parameters of dyslexic students improve with age, this improvement is slower than typical students for the age groups considered. However, this is expected to change later as the reading ability of typical students reaches a plateau. Towards the end of primary school, dyslexic students seem to cope better with silent reading, and even better towards secondary and upper secondary grades, while this is achieved in the 3^rd^ and 4^th^ grade for typical students. Hence, it seems that in most cases dyslexic students can master both loud and silent reading sufficiently, they only need more time.

Overall, our research indicates the typical readers have better values in eye-tracking parameters than dyslexics, in both silent and loud reading. Despite the preferences that typical and dyslexic students might have, those with dyslexia face greater difficulties in both reading modes when compared to typical readers of similar age. These difficulties have been noticed in every “word-based” or “non word-based” parameter examined here).

There are some exceptions to what was previously stated, which is that typical readers prefer to read silently, while those with dyslexia prefer loud reading. In particular, there are a few typical students (less than 5% of the overall typical population) that had positive asymmetries i.e., they preferred to read out loud. In some cases, we assume that this might be due to not fully developed learning skills based on the age of students, and hence the lack of training in silent reading. Such discrepancies may also be due to personal preference towards loud reading, or to atypical educational training. Moreover, a few dyslexic students show a preference for silent reading (less than 3% of the overall population). This could be explained by other co-existing articulation difficulties with dyslexia (2). In this case, the struggling performance of dyslexic students is mainly manifested in loud reading, which makes silent reading the preferred mode.

### Conclusion

In conclusion, our data suggest that Greek students have different preferences in reading mode, silent or loud, based on the difficulties they face in reading and on their age and grade. In particular, dyslexic students tend to prefer loud reading, while typical students prefer silent reading. Importantly, our research results provide evidence that dyslexic students improve their reading ability over the years, even though their improvement rate is lower than that of typical students. Some students with dyslexia have the ability to become good readers, but they need more time, more effort, appropriate intervention and support. In addition, we conclude that eye-tracking technology can provide quantitative data on both loud and silent reading and can become a valuable additional tool in the hands of the experts, allowing personal assessment and long-term follow up of reading skills. Future research could pursue further the implications of these findings and examine the possibility that silent and loud reading evaluations are important for the evaluation of dyslexia.

### Acknowledgment

This research has been co‐financed by the European Union and Greek national funds through the Operational Program Competitiveness, Entrepreneurship and Innovation, under the call RESEARCH – CREATE – INNOVATE (project code: T1EDK-00858). The authors Ioannis Smyrakis and Vassilios Andreadakis are affiliated to Optotech Ltd. which provided support in the form of salary. Ioannis M. Aslanides is affiliated to Optotech Ltd. in the form of ownership of the company

### supplementary-material



## References

[b1] Aaron, P. G., Baxter, C. F., & Lucenti, J. (1980). Developmental dyslexia and acquired alexia: Two sides of the same coin? Brain and Language, 11(1), 1–11. 10.1016/0093-934X(80)90104-2 0093-934X 7427710

[b2] Alves, L. M., Reis, C., & Pinheiro, Â. (2015). Prosody and reading in dyslexic children. Dyslexia (Chichester, England), 21(1), 35–49. 10.1002/dys.1485 1099-0909 25363804

[b3] APA. A. P. A. (2013). DSM-V Diagnostic and statistical manual of mental disorders. American Psychiatric Association(Arlington, VA,).

[b4] Bilbao, C., & Piñero, D. P. (2020). Diagnosis of oculomotor anomalies in children with learning disorders. Clinical & Experimental Optometry, 103(5), 597–609. 10.1111/cxo.13024 1444-0938 31869866

[b5] Blythe, H., & Joseph, H. (2011). Children’s eye movements during reading (L. S., I. G. I., & E. S. Eds.): Oxford University Press.

[b6] Borleffs, E., Maassen, B. A. M., Lyytinen, H., & Zwarts, F. (2019). Cracking the code: The impact of orthographic transparency and morphological-syllabic complexity on reading and developmental dyslexia. Frontiers in Psychology, 9, 2534. 10.3389/fpsyg.2018.02534 1664-1078 30662416 PMC6328448

[b7] Bucci, M. P., Vernet, M., Gerard, C.-L., & Kapoula, Z. (2009). Normal speed and accuracy of saccade and vergence eye movements in dyslexic reader children. Journal of Ophthalmology, 2009. 10.1155/2009/325214 2090-0058 20309415 PMC2836913

[b8] Burge, P. D. (1983). Comprehension and rate: Oral vs. silent reading for low achievers. Reading Horizons: A Journal of Literacy and Language Arts, 23(3), 11.

[b9] Buswell, G. T. (1921). The relationship between eye-perception and voice-response in reading. Journal of Educational Psychology, 12(4), 217–227. 10.1037/h0070548 1939-2176

[b10] Caldani, S., Gerard, C.-L., Peyre, H., & Bucci, M. P. (2020). Pursuit eye movements in dyslexic children: Evidence for an immaturity of brain oculomotor structures? Journal of Eye Movement Research, 13(1). 10.16910/jemr.13.1.5 1995-8692 33828780 PMC7881873

[b11] Caldani, S., Gerard, C.-L., Peyre, H., & Bucci, M. P. (2020). Visual attentional training improves reading capabilities in children with dyslexia: An eye tracker study during a reading task. Brain Sciences, 10(8), 558. 10.3390/brainsci10080558 2076-3425 32824168 PMC7464337

[b12] Carlisle, J. F., & Stone, C. A. (2005). Exploring the role of morphemes in word reading. Reading Research Quarterly, 40(4), 428–449. 10.1598/RRQ.40.4.3 0034-0553

[b13] De Luca, M., Borrelli, M., Judica, A., Spinelli, D., & Zoccolotti, P. (2002). Reading words and pseudowords: An eye movement study of developmental dyslexia. Brain and Language, 80(3), 617–626. 10.1006/brln.2001.2637 0093-934X 11896661

[b14] De Luca, M., Pontillo, M., Primativo, S., Spinelli, D., & Zoccolotti, P. (2013). The eye-voice lead during oral reading in developmental dyslexia. Frontiers in Human Neuroscience, 7, 77-93. 10.3389/fnhum.2013.00696 1662-5161 24223541 PMC3818695

[b15] Di Noto, P., Uta, S., & DeSouza, J. F. (2013). Eye exercises enhance accuracy and letter recognition, but not reaction time, in a modified rapid serial visual presentation task. PLoS One, 8(3), e59244. 10.1371/journal.pone.0059244 1932-6203 23527146 PMC3602039

[b16] Fairbanks, G. (1937). The relation between eye-movements and voice in the oral reading of good and poor silent readers. Psychological Monographs, 48(3), 78–107. 10.1037/h0093394 0096-9753

[b17] FragaGonzález, G., Karipidis, I. I., & Tijms, J. (2018). Dyslexia as a neurodevelopmental disorder and what makes it different from a chess disorder. Brain Sciences, 8(10), 189. 10.3390/brainsci8100189 2076-3425 30347764 PMC6209961

[b18] Gagliano, A., Ciuffo, M., Ingrassia, M., Ghidoni, E., Angelini, D., Benedetto, L., Germanò, E., & Stella, G. (2015). Silent reading fluency: Implications for the assessment of adults with developmental dyslexia. Journal of Clinical and Experimental Neuropsychology, 37(9), 972–980. 10.1080/13803395.2015.1072498 1744-411X 26332176

[b19] Hale, A. D., Skinner, C. H., Williams, J., Hawkins, R., Neddenriep, C. E., & Dizer, J. (2007). Comparing comprehension following silent and aloud reading across elementary and secondary students: Implication for curriculum-based measurement. The Behavior Analyst Today, 8(1), 9–23. 10.1037/h0100101 1539-4352

[b20] Hampson, M., Tokoglu, F., Sun, Z., Schafer, R. J., Skudlarski, P., Gore, J. C., & Constable, R. T. (2006). Connectivity-behavior analysis reveals that functional connectivity between left BA39 and Broca’s area varies with reading ability. NeuroImage, 31(2), 513–519. 10.1016/j.neuroimage.2005.12.040 1053-8119 16497520

[b21] Hutzler, F., & Wimmer, H. (2004). Eye movements of dyslexic children when reading in a regular orthography. Brain and Language, 89(1), 235–242. 10.1016/S0093-934X(03)00401-2 0093-934X 15010255

[b22] Jafarlou, F., Jarollahi, F., Ahadi, M., & Sadeghi-Firoozabadi, V. (2020). Effects of oculomotor rehabilitation on the cognitive performance of dyslexic children with concurrent eye movement abnormalities. Early Child Development and Care, 1–13. 10.1080/03004430.2020.1793759 0300-4430

[b23] Järvilehto, T., Nurkkala, V.-M., & Koskela, K. (2009). The role of anticipation in reading. Pragmatics & Cognition, 17(3), 509–526. 10.1075/pc.17.3.02jar 0929-0907

[b24] Johnstone, J., Galin, D., Fein, G., Yingling, C., Herron, J., & Marcus, M. (1984). Regional brain activity in dyslexic and control children during reading tasks: Visual probe event-related potentials. Brain and Language, 21(2), 233–254. 10.1016/0093-934X(84)90049-X 0093-934X 6704700

[b25] Juel, C., & Holmes, B. (1981). Oral and silent reading of sentences. Reading Research Quarterly, 16, 545–568. 10.2307/747315 0034-0553

[b26] Kirby, P. (2020). Dyslexia debated, then and now: A historical perspective on the dyslexia debate. Oxford Review of Education, 46(4), 472–486. 10.1080/03054985.2020.1747418 0305-4985 32939102 PMC7455059

[b27] Kirkby, J. A., Webster, L. A. D., Blythe, H. I., & Liversedge, S. P. (2008). Binocular coordination during reading and non-reading tasks. Psychological Bulletin, 134(5), 742–763. 10.1037/a0012979 0033-2909 18729571

[b28] Korneev, A., Matveeva, E. Y., & Akhutina, T. (2017). Silent reading in Russian primary schoolchildren: An eye tracking study. Psikhologiya, 14, 219.

[b29] Krieber, M., Bartl-Pokorny, K. D., Pokorny, F. B., Zhang, D., Landerl, K., Körner, C., Pernkopf, F., Pock, T., Einspieler, C., & Marschik, P. B. (2017). Eye movements during silent and oral reading in a regular orthography: Basic characteristics and correlations with childhood cognitive abilities and adolescent reading skills. PLoS One, 12(2), e0170986. 10.1371/journal.pone.0170986 1932-6203 28151950 PMC5289712

[b30] Lyon, G. R., Shaywitz, S., & Shaywitz, B. (2003). A definition of dyslexia. Annals of Dyslexia, 53(1), 1–14. 10.1007/BF02648210 0736-9387 24234186

[b31] Mani, N., & Huettig, F. (2014). Word reading skill predicts anticipation of upcoming spoken language input: A study of children developing proficiency in reading. Journal of Experimental Child Psychology, 126, 264–279. 10.1016/j.jecp.2014.05.004 1096-0457 24955519

[b32] Martos, F., & Vila, J. (1990). Differences in eye movements control among dyslexic, retarded and normal readers in the Spanish population. Reading and Writing, 2(2), 175–188. 10.1007/BF00401801 0922-4777

[b33] Nation, K. (2019). Children’s reading difficulties, language, and reflections on the simple view of reading. Australian Journal of Learning Difficulties, 24(1), 47–73. 10.1080/19404158.2019.1609272 1940-4158

[b34] Polychroni, F. (2001). Specific Learning Difficulties. Pedio.

[b35] Protopapas, A., & Vlahou, E. L. (2009). A comparative quantitative analysis of Greek orthographic transparency. Behavior Research Methods, 41(4), 991–1008. 10.3758/BRM.41.4.991 1554-3528 19897808

[b36] Radach, R., & Kennedy, A. (2013). Eye movements in reading: Some theoretical context. Quarterly Journal of Experimental Psychology, 66(3), 429–452. 10.1080/17470218.2012.750676 1747-0226 23289943

[b37] Rayner, K. (1998). Eye movements in reading and information processing: 20 years of research. Psychological Bulletin, 124(3), 372–422. 10.1037/0033-2909.124.3.372 0033-2909 9849112

[b38] Rayner, K., Chace, K. H., Slattery, T., & Ashby, J. (2009). Eye movements as reflections of comprehension processes in reading. Scientific Studies of Reading, 10(3), 241–255. 10.1207/s1532799xssr1003_3 1088-8438

[b39] Siok, W. T., Spinks, J. A., Jin, Z., & Tan, L. H. (2009). Developmental dyslexia is characterized by the co-existence of visuospatial and phonological disorders in Chinese children. Current Biology, 19(19), R890–R892. 10.1016/j.cub.2009.08.014 1879-0445 19825347

[b40] Snowling, M. J. (1983). The comparison of acquired and developmental disorders of reading: A discussion. 10.1016/0010-0277(83)90027-6 6685010

[b41] Snowling, M. J., Hulme, C., & Nation, K. (2020). Defining and understanding dyslexia: Past, present and future. Oxford Review of Education, 46(4), 501–513. 10.1080/03054985.2020.1765756 0305-4985 32939103 PMC7455053

[b42] Sprenger-Charolles, L., Siegel, L., Jimenez, J., & Ziegler, J. (2011). Prevalence and reliability of phonological, surface, and mixed profiles in dyslexia: A review of studies conducted in languages varying in orthographic depth. Scientific Studies of Reading, 15(6), 498–521. 10.1080/10888438.2010.524463 1088-8438

[b43] Stein, J. (2018). What is developmental dyslexia? Brain Sciences, 8(2), 26. 10.3390/brainsci8020026 2076-3425 29401712 PMC5836045

[b44] Talli, I., Sprenger-Charolles, L., & Stavrakaki, S. (2016). Specific language impairment and developmental dyslexia: What are the boundaries? Data from Greek children. Research in Developmental Disabilities, 49-50, 339–353. 10.1016/j.ridd.2015.12.014 1873-3379 26773216

[b45] Taylor, S. E. (2006). Fluency in silent reading. Retrieved January, 15, 2007.

[b46] Thiagarajan, P., Ciuffreda, K. J., Capo-Aponte, J. E., Ludlam, D. P., & Kapoor, N. (2014). Oculomotor neurorehabilitation for reading in mild traumatic brain injury (mTBI): An integrative approach. NeuroRehabilitation, 34(1), 129–146. 10.3233/NRE-131025 1878-6448 24284470

[b47] Tobii. (2016). Tobii 4c Eye Tracker. Retrieved from https://help.tobii.com/hc/en-us/articles/213414285-Specifications-for-the-Tobii-Eye-Tracker-4C

[b48] Vorstius, C., Radach, R., & Loniganb, J. C. (2014). Eye movements in developing readers: A comparison of silent and oral sentence reading. Visual Cognition, 22(3-4), 458–485. 10.1080/13506285.2014.881445 1350-6285

