# Supplementary material for: Silent versus Reading Out Loud modes: An eye-tracking study
Source: J Eye Mov Res. 2021 Oct 21;14(2):10.16910/jemr.14.2.1. doi: 10.16910/jemr.14.2.1 (PMC8565638; doi:10.16910/jemr.14.2.1)
Supplement: Supplementary file 1 [file jemr-14-02-a-SD1-01.pdf]

Silent versus Reading Out Loud modes: an eye-tracking study

Supplementary tables

Table 1: p values of all asymmetries of "non word- based" parameters for both populations

	Asymmetries						
Typical	Reading speed	Fix. no	Mean fix. dur.	Mean sac. len	25% perc.	50% perc.	75% perc.
Group A	0.2434	0.0046	0.0157	0.2171	0.1710	0.3716	0.1262
Group B	0.0091	0.2828	0.0005	0.0013	0.0585	0.0065	0.0004
Group C	0.0652	0.3737	0.0021	0.0295	0.1602	0.0607	0.0054
Dyslexic	Reading speed	Fix. no	Mean fix. dur.	Mean sac. len	25%	50%	75%
Group A	0.0003	0.0009	0.0162	0.0001	0.0011	0.0000	0.0008
Group B	0.0002	0.0017	0.0702	0.2477	0.0133	0.0562	0.2295
Group C	0.4790	0.1894	0.3648	0.3326	0.2046	0.1128	0.4324

Table 2: p values of all asymmetries of "word-based" parameters for both populations

	Asymmetries				
Typical	No fix. words	Mult. fix. words	Gaze dur 6-7	Gaze dur 8+	Back refix.
Group A	0.1724	0.1863	0.1835	0.0688	0.0001
Group B	0.0034	0.0667	0.0654	0.0452	0.1122
Group C	0.0037	0.4297	0.0236	0.0101	0.3089
Dyslexic	No fix. words	Mult. fix. words	Gaze dur 6-7	Gaze dur 8+	Back refix.
Group A	0.0541	0.0091	0.0034	0.0002	0.0130
Group B	0.4164	0.0459	0.1738	0.0174	0.0539
Group C	0.0970	0.3108	0.0180	0.0458	0.0928

Table 3: The ratios d_T and d_D for all parameters

	Ratios						
	Reading speed	Fix. no	Mean fix. dur.	Mean sac. len	25% perc.	50% perc.	75% perc.
d_T Group A	0,06	0,17	0,11	0,08	0,16	0,04	0,11
d_T Group B	0,31	0,04	0,40	0,59	0,45	0,57	0,56
d_T Group C	0,19	0,03	0,39	0,33	0,22	0,41	0,40
	Reading speed	Fix. no	Mean fix. dur.	Mean sac. len	25%	50%	75%
d_D Group A	0,22	0,39	0,18	0,30	0,34	0,39	0,19
d_D Group B	0,15	0,24	0,12	0,05	0,23	0,10	0,05
d_D Group C	0,01	0,17	0,06	0,06	0,14	0,08	0,03

Table 3: continue

	Ratios				
	No fix. words	Mult. fix. words	Gaze dur 6-7	Gaze dur 8+	Back refix.
d_T Group A	0,12	0,10	0,04	0,07	0,43
d_T Group B	0,50	0,14	0,11	0,12	0,09
d_T Group C	0,30	0,02	0,23	0,16	0,04
	No fix. words	Mult. fix. words	Gaze dur 6-7	Gaze dur 8+	Back refix.
d_D Group A	0,21	0,21	0,31	0,37	0,52
d_D Group **B**	0,02	0,13	0,09	0,26	0,27
d_D Group **C**	0,17	0,08	0,38	0,23	0,34

Table 4: The differences D, which are the absolute differences between typical and dyslexic parameter values

	Differences D						
	Reading speed	Fix. no	Mean fix. dur.	Mean sac. len	25% perc.	50% perc.	75% perc.
Group A	0,06	135,21	58,36	36,85	18,62	32,28	54,47
Group B	0,31	121,86	41,16	46,54	28,45	44,76	66,07
Group C	0,19	132,73	38,03	55,72	42,57	57,60	68,38

Table 4: continue

	Differences D				
	No fix. words	Mult. fix. words	Gaze dur 6-7	Gaze dur 8+	Back refix.
Group A	8,85	21,50	310,29	471,95	19,15
Group B	14,36	24,76	164,35	238,05	22,95
Group C	23,60	37,39	148,22	240,31	21,08
